# Two new species of the genus *Epuraea* Erichson, 1843 from China (Coleoptera, Nitidulidae, Epuraeinae)

**DOI:** 10.3897/zookeys.445.7163

**Published:** 2014-10-13

**Authors:** Mengjiao Zhao, Min Huang, Xingke Yang, Alexander G. Kirejtshuk

**Affiliations:** 1 Key Laboratory of Plant Protection Resources and Pest Management of Ministry of Education, Entomological Museum, Northwest A&F University, Yangling, Shaanxi 712100, China; 2 Key Laboratory of Zoological Systematics and Evolution, Institute of Zoology, Chinese Academy of Sciences, Beijing 100101, China; 3 Zoological Institute RAS, Universitetskaya nab., 1, 199034 St. Petersburg, Russia; 4 CNRS UMR 5202, Muséum National d’Histoire Naturelle, CP 50, Entomologie, 45, rue Buffon, F- 75005 Paris, France

**Keywords:** Coleoptera, Nitidulidae, *Epuraea*, *Micruria*, new species, China, Sichuan

## Abstract

Two new species belonging to the *consobrina*-group of the subgenus *Micruria* Reitter, 1875 (genus *Epuraea* Erichson, 1843), Epuraea (Micruria) lanuginosa **sp. n.** and Epuraea (Micruria) pulliginis **sp. n.**, found in Sichuan Province, China, are described. Pictures and details of structures important for diagnostics of the new species, including external characters and genitalia are given.

## Introduction

The *Micruria* Reitter, 1875 is recognized as a taxon with a subgeneric status (Reitter 1884a, 1884b; Grouvelle 1908; Spornraft 1966; Jelínek 1978; Hisamatsu 1985; Kirejtshuk 1992, 1998). Reitter (1875) included in it the following species: *Micruria japonica* Reitter, 1875, *Micruria mandiularis* Reitter, 1873, *Micruria nitida* Reitter, 1875 and *Micruria macrophthalma* Reitter, 1875. Additional species attributed to *Micruria* were later described by Grouvelle (1892, 1894, 1897, 1902, 1908, 1914), Hisamatsu (1961), Jelínek (1978) and Kirejtshuk (1992, 1997, 1998, 2005). According to Kirejtshuk (1998) the subgenus *Micruria* can be divided into five groups of species: *mandibularis*-group, *auripubens*-group, *melanocephala*-group, *grouvellei*-group, and *consobrina*-group. There are 55 known species spread in the Eastern Hemisphere, mainly in the Palaearchiarctic (East Chinese) Province and the Indo-Malayan Region, of which 16 were recorded from China (Kirejtshuk 1998). Here we add two new species, Epuraea (Micruria) lanuginosa sp. n. and Epuraea (Micruria) pulliginis sp. n., which are placed in the *consobrina*-group.

Most members of *Micruria* occur mainly in mountain forests. Some species have been found under bark with fermenting sap or oozing cambial tissue, in decomposing grass or leaves, and similar substrates of plant origin. However, adults of many representatives are associated with flowers of trees and bushes in nemoral forests (Kirejtshuk 1998).

## Material and methods

The holotypes and paratypes of the new species are deposited in the collection of Northwest A&F University (NWUAF), Yangling and the Institute of Zoology, Chinese Academy of Sciences (IZCAS), Beijing.

All descriptions and measurements were made under an Olympus SZX 10 microscope. Figures were made using Leica ZOOM 2000 microscope with an O-image CCD. Images were produced using the software Synoptic Automontage.

## Taxonomic treatment

### Family Nitidulidae Latreille, 1802 Subfamily Epuraeinae Kirejtshuk, 1986 Tribe Epuraeini Kirejtshuk, 1986 Genus *Epuraea* Erichson, 1843

#### Subgenus Micruria

Taxon classificationAnimaliaColeopteraNitidulidae

Reitter, 1875

##### Type species.

*Epuraea mandibularis* Reitter, 1873, designated by Kirejtshuk (1998).

#### Epuraea (Micruria) lanuginosa

Taxon classificationAnimaliaColeopteraNitidulidae

Zhao, Huang & Kirejtshuk sp. n.

http://zoobank.org/E3D2A67E-6AED-440F-B00F-3FEE2047E06F

Figs 1 –8

##### Type material.

**Holotype.** ♂, China: Sichuan, Pingwu, Laohegou; 1800m, 7.VII.2013, Lingling Ren leg. (NWSUAF). **Paratypes.** (1♂, 3♀), same data as holotype (NWSUAF).

##### Description.

**Figures 1, 2.**
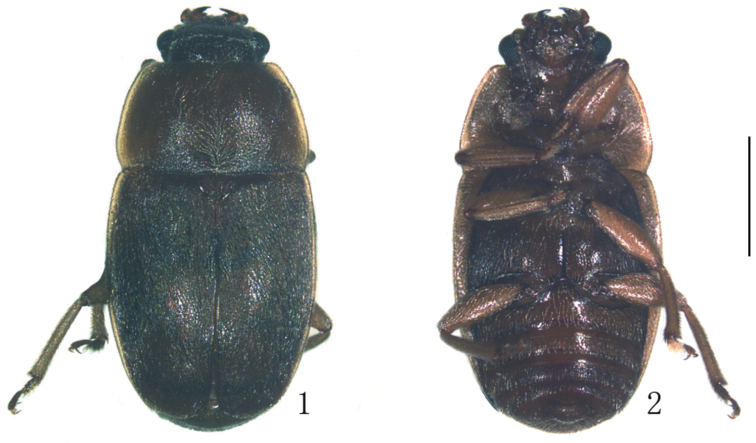
Epuraea (Micruria) lanuginosus sp. n. male. **1** habitus, dorsal view **2** same, ventral view. Scale bar = 1 mm.

*Body.* Length 3.7 mm, breadth 1.7 mm, height 0.9 mm. Oblong, moderately convex; dorsum dark brown and with bronze lustre, underside reddish-brown with appendages slightly lighter, pronotal and elytral margins light reddish to yellow; dorsum with long, strongly conspicuous and sparse silver yellowish hairs, which are three times longer than distance between their insertions (Figs 1, 2).

*Integument.* Head with irregular and indistinct punctures, surface between them microreticulated. Pronotum with finer punctures nearly as large as eye facets; interspaces between them greater than a puncture diameter and smoothly microreticulated. Scutellum triangular with shallow punctures smaller than punctures on pronotum and interspaces among them equal to a puncture diameter or greater. Elytra with punctures slightly smaller than those on scutellum, interspaces among them greater than a puncture diameter and microreticulated. Pygidial surface nearly as that of elytra, but with shallower punctures and denser pubescence. Abdominal ventrites with moderately distinct punctures slightly smaller than eye facets in diameter, interspaces among them smoothly microreticulated.

**Figures 3–8.**
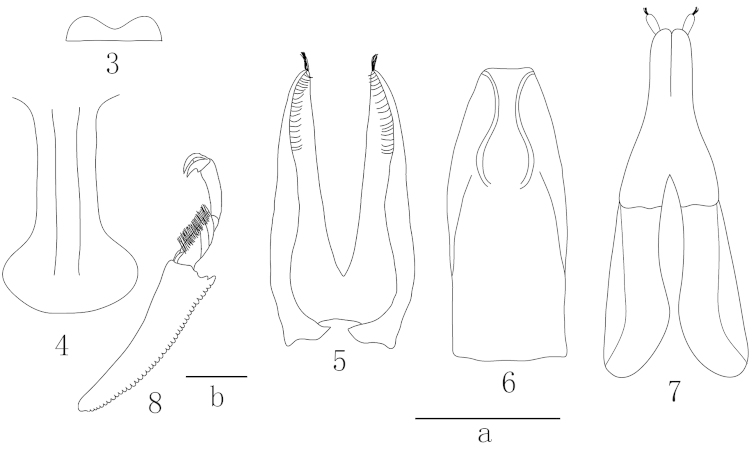
Epuraea (Micruria) lanuginosus sp. n. **3** labrum, dorsal view **4** prosternal process, ventral view **5** tegmen, ventral view **6** penis trunk, dorsal view **7** ovipositors, ventral view **8** protibia, dorsal view. Scale bars = 0.2 mm: **a** for Figs **3**–**7**, **b** for Fig. **8.**

*Head.* Head slightly convex and eyes medium-sized. Labrum with a shallow median incision (Fig. 3). Antennal grooves start from hypostomal sinuses and are convergent posteriorly. Ultimate labial palpomere approximately 3 times as long as thick and somewhat narrowed at apex. Antennae slightly longer than head width, club approximately 2/5 of total length and about 1.5 times as long as wide. Pronotum moderately convex and 1.8 times as broad as long with apex emarginate, base lightly sinuate near posterior angles, sides arcuate with margins subexplanate and somewhat translucent, anterior angles square and posterior ones projecting slightly; widest at posterior angles, narrowed to both base and apex. Prosternal process curved along procoxae, widened apically (Fig. 4). Elytra much longer than their combined width (1.3:1), their sides arcuate and margins narrower than pronotum, with separately rounded apices, leaving uncovered the pygidium and part of preceding tergite. Pygidium triangular, apex of anal sclerite exposed from under pygidium. Distance between mesocoxae as great as width of antennal club and distance between metacoxae about three times as great as that between mesocoxae. Elytral epipleura at base as wide as antennal club. Metaventrite slightly convex with a distinct median depression.

*Legs.* All tibiae narrow and long; protibia with teeth gradually increasing in size along outer edge and two distinct larger teeth at apex. Mesotibia slightly curved inside near apex; tarsal claws with strong teeth at base (Fig. 8).

*Aedeagus.* Tegmen and penis trunk moderately sclerotized (Figs 5, 6).

##### Female.

The apex of mesotibiae not curved. Ovipositor moderately long and weakly sclerotized (Fig. 7).

##### Etymology.

The name derives from the conspicuously pubescent dorsum of the species (‘*lanuginosus*’ in Latin means ‘woolly’, ‘downy’).

##### Notes.

Having moderately convex body, comparatively distinct dorsal punctation, subexplanate pronotal sides, simple mesotibiae, truncate apex of penis trunk the new species seems to belong to the *consobrina*-group which is hitherto known to comprise the following species: Epuraea (Micruria) bergeri Sjöberg, 1939; Epuraea (Micruria) consobrina Grouvelle, 1892; Epuraea (Micruria) kompantzevi Kirejtshuk, 1999; Epuraea (Micruria) pulliginis sp. n.; Epuraea (Micruria) reticulata Grouvelle, 1892, Epuraea (Micruria) scapha Kirejtshuk, 1999, Epuraea (Micruria) subita Kirejtshuk, 1999 and Epuraea (Micruria) subreticulata Grouvelle, 1892. It can be easily distinguished from all the members of the group in the bronze lustre on its rather dark dorsum, deep narrow depression along the middle of metaventrite and peculiar structure of aedeagus. Besides, it differs from:

- Epuraea (Micruria) bergeri in the less convex pronotum narrowed at base and with more shallowly emarginate anterior edge and more clearly explanate and translucent sides, elytra more narrowing towards transversely oblique apices (not transverse), rounded apex of prosternal process, simple metafemur and metatibia, strong tooth at base of tarsal claws, ovipositor with wider base of coxites;
- Epuraea (Micruria) consobrina in the subunicolorous disks of pronotum and elytra, coarser and deeper punctation (particularly on elytra), longer and denser silver pubescence, narrower explanate stripes of elytra, obliquely rounded elytral apices (not obliquely truncate), rounded apex of prosternal process, strong tooth at base of tarsal claws, narrower ovipositor with shorter coxites;
- Epuraea (Micruria) kompantzevi in the more slender (not subovoid) body, denser and more clear dorsal punctation, pronotum narrowing at base, less gently sloping pronotal and elytral sides, subtruncate elytral apices (never forming a join curve), projecting subapical teeth of protibiae, lack of sexual dimorphism in elytral apices;
- Epuraea (Micruria) pulliginis sp. n. in the much denser dorsal punctation, silver pubescence, elytra less narrowing towards subtruncate apices, more projecting subapical teeth on protibiae, narrower coxites of ovipositor;
- Epuraea (Micruria) reticulata in the subunicolorous dorsum, much denser and more distinct dorsal punctation, denser and more conspicuous dorsal pubescence, widely rounded lobes of labrum, elytra more narrowing towards transversely oblique apices (not transverse), simple male metafemora, projecting subapical teeth of protibiae;
- Epuraea (Micruria) scapha in the much more slender body, denser and more clear dorsal punctation, less gently sloping pronotal and elytral sides, subtruncate elytral apices (not forming a join curve), projecting subapical teeth of protibiae, simple male metafemora, meso- and metatibiae, and lack of sexual dimorphism in elytral apices, ovipositor with coxites shorter and narrower at base;
- Epuraea (Micruria) subita in the less convex body and particularly pronotum with more clearly explanate and translucent sides, rounded apex of prosternal process, more elytra narrowing towards transversely oblique apices (not transverse), simple metafemur, strong tooth at base of tarsal claws, ovipositor with wider base of coxites.

#### Epuraea (Micruria) pulliginis

Taxon classificationAnimaliaColeopteraNitidulidae

Zhao, Huang & Kirejtshuk sp. n.

http://zoobank.org/77C35622-431E-4979-B47C-5AE642AEA2F6

Figs 9 –16

##### Type material.

**Holotype.** ♂, China, Sichuan, Wolong, 2200-2600m, 29.VII.1983, Xuezhong, Zhang leg., (IZCAS). **Paratypes.** 1♂,7♀, same data as holotype (IZCAS).

##### Description.

**Figures 9, 10.**
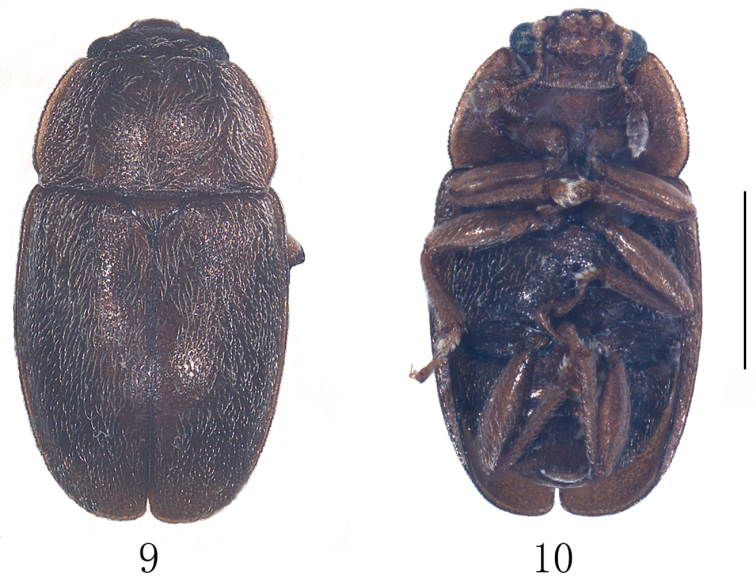
Epuraea (Micruria) pulliginis sp. n., male. **9** habitus, dorsal view **10** same, ventral view. Scale bar = 1 mm.

*Body.* Length 3.2 mm, breadth 1.7 mm, hight 0.8 mm. Body oval, rather convex dorsally, dorsum nearly unicoloured chestnut brown with lighter pronotal sides, underside dark brown with brown appendages and prosternum. Pubescence silver, closely adpressed, and equal to or somewhat longer than distance between their insertions (Figs 9, 10).

*Integument.* Head with irregularly sized and spaced punctures. Pronotum with moderately deep punctation nearly as large as eye facets in diameter, interspaces between them slightly greater than one puncture diameter, surface microreticulated; elytra with slightly coarse punctures less than eye facets in diameter, interspaces between them approximately twice as great as a puncture diameter. Metaventrite and abdominal ventrites with indistinct punctures and microreticulated.

**Figures 11–16.**
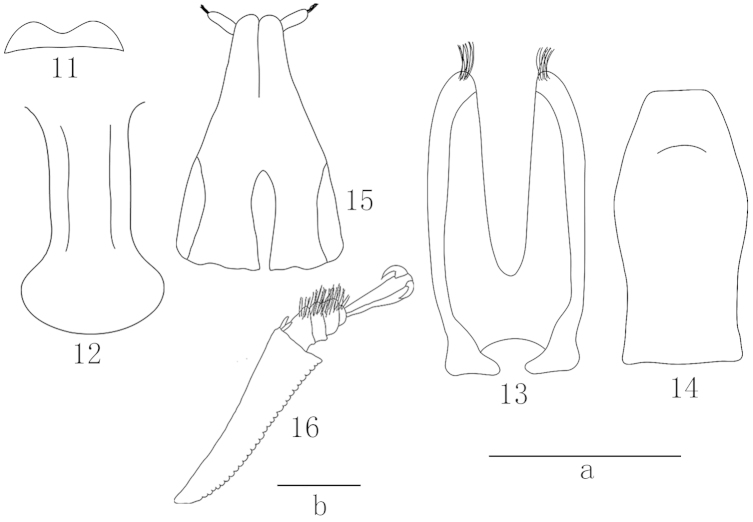
Epuraea (Micruria) pulliginis sp. n. **11** prosternal process, ventral view **12** labrum, dorsal view **13** tegmen, ventral view **14** penis trunk, dorsal view **15** apex of ovipositor, ventral view **16** protibia, left view. Scale bar = 0.2 mm; **a** for Figs **11**–**15**, **b** for Fig. **16.**

*Head* short, half as long as the distance between eyes (consisting of moderately fine facets with greater diameter than that of punctures). Labrum with shallow emargination in the middle (Fig. 11). Ultimate labial palpomere approximately three times as long as thick and somewhat narrower at apex. Antennae markedly longer than head breadth, antennal club oval, and composing approximately 1/3 of total length. Pronotum evenly convex, 1.8 times as wide as long, with apex transverse, base slightly sinuate near angles, sides arcuate and margins narrowly explanate, anterior angles projecting and posterior angles obtuse; widest just at posterior angles. Prosternal process moderately curved along procoxae, moderately widened apically (Fig. 12). Elytra much longer than combined width, gradually narrowing to rounded apices, sides arcuate, and margins subexplanate and somewhat translucent. Pygidium not exposed from under elytral apices. Distance between procoxae subequal and that between matacoxae nearly three times more than between mesocoxae. Epipleura slightly narrower than antennal club.

*Legs.* All legs long and narrow. Protibia (Fig. 16) wider than meso- and metatibiae, with gradually increasing teeth along outer edges and two subapical long spurs. Protarsi 4/5 as wide as corresponding tibiae, meso- and metatarsi much narrower. Tarsal claws with strong teeth at base (Fig. 16).

*Aedeagus.* Tegmen well sclerotized, penis trunk moderately sclerotized (Figs 13, 14).

##### Female.

Ovipositor simple, short and weakly sclerotized (Fig. 15).

##### Etymology.

The specific epithet emphsizes the brown coloration of the species (Latin “*pulliginis*” – singular, genitive case from “*pulligo*” – brown, dark color).

##### Notes.

This new species appears to be closely related to Epuraea (Micruria) kompantzevi (*consobrina*-group) differing from it by having more slender (not subovoid) body, denser and more clear dorsal punctation, more conspicuous pubescence, less gently sloping pronotal and elytral sides, subtruncate elytral apices (not forming a join curve), projecting subapical teeth of protibiae, lack of sexual dimorphism in elytral apices and peculiar structure of the aedeagus. Besides, in addition of characteristic structure of male genitalia of Epuraea (Micruria) pulliginis sp. n. differs from:

- Epuraea (Micruria) bergeri in the pronotum narrowed at base, elytra more narrowing towards transversely oblique apices (not transverse), rounded apex of prosternal process, simple metafemur and metatibia, strong tooth at base of tarsal claws, ovipositor with wider base of coxites;
- Epuraea (Micruria) consobrina in the subunicolorous disks of pronotum and elytra, coarser and deeper punctation (particularly on elytra), longer and denser silver pubescence, narrower explanate stripes of elytra, obliquely rounded elytral apices (not obliquely truncate), rounded apex of prosternal process, strong tooth at base of tarsal claws, narrower ovipositor with shorter coxites;
- Epuraea (Micruria) pulliginis sp. n. in the lighter coloration without bronze shine on dorsum, much sparser dorsal punctation, golden pubescence, elytra more narrowing towards subtruncate apices and completely covering abdomen, lack of deepened narrow nedian depression along the middle of metaventrite, less projecting subapical teeth on protibiae, wider coxites of ovipositor;
- Epuraea (Micruria) reticulata in the subunicolorous dorsum, much denser and more distinct dorsal punctation, denser and more conspicuous dorsal pubescence, widely rounded lobes of labrum, elytra more narrowing towards transversely oblique apices (not transverse), simple male metafemora, projecting subapical teeth of protibiae;
- Epuraea (Micruria) scapha in the much more slender body, denser and more clear dorsal punctation, less gently sloping pronotal and elytral sides, obliquely subtruncate elytral apices (not forming a join curve), projecting subapical teeth of protibiae, simple male metafemora, meso- and metatibiae, and lack of sexual dimorphism in elytral apices, ovipositor with coxites shorter and narrower at base;
- Epuraea (Micruria) subita in the less convex body, rounded apex of prosternal process, elytra narrowing towards transversely oblique apices (not transverse), simple metafemur, strong tooth at base of tarsal claws, ovipositor with wider base of coxites.

## Supplementary Material

XML Treatment for Micruria

XML Treatment for Epuraea (Micruria) lanuginosa

XML Treatment for Epuraea (Micruria) pulliginis
